# Deep Gated Hebbian Predictive Coding Accounts for Emergence of Complex Neural Response Properties Along the Visual Cortical Hierarchy

**DOI:** 10.3389/fncom.2021.666131

**Published:** 2021-07-28

**Authors:** Shirin Dora, Sander M. Bohte, Cyriel M. A. Pennartz

**Affiliations:** ^1^Cognitive and Systems Neuroscience Group, Swammerdam Institute for Life Sciences, University of Amsterdam, Amsterdam, Netherlands; ^2^Intelligent Systems Research Centre, Ulster University, Londonderry, United Kingdom; ^3^Machine Learning Group, Centre of Mathematics and Computer Science, Amsterdam, Netherlands

**Keywords:** visual processing, predictive coding, deep biologically plausible learning, selectivity, sparseness, sensory neocortex, inference, representation learning

## Abstract

Predictive coding provides a computational paradigm for modeling perceptual processing as the construction of representations accounting for causes of sensory inputs. Here, we developed a scalable, deep network architecture for predictive coding that is trained using a gated Hebbian learning rule and mimics the feedforward and feedback connectivity of the cortex. After training on image datasets, the models formed latent representations in higher areas that allowed reconstruction of the original images. We analyzed low- and high-level properties such as orientation selectivity, object selectivity and sparseness of neuronal populations in the model. As reported experimentally, image selectivity increased systematically across ascending areas in the model hierarchy. Depending on the strength of regularization factors, sparseness also increased from lower to higher areas. The results suggest a rationale as to why experimental results on sparseness across the cortical hierarchy have been inconsistent. Finally, representations for different object classes became more distinguishable from lower to higher areas. Thus, deep neural networks trained using a gated Hebbian formulation of predictive coding can reproduce several properties associated with neuronal responses along the visual cortical hierarchy.

## Introduction

According to classical neurophysiology, perception is thought to be based on sensory neurons which extract knowledge from the world by detecting objects and features, and report these to the motor apparatus for behavioral responding (Barlow, 1953; Lettvin et al., 1959; Riesenhuber and Poggio, 1999). This doctrine is radically modified by the proposal that percepts of objects and their features are representations constructed by the brain in attempting to account for the causes underlying sensory inputs (Kant, 1781; von Helmholtz, 1867; Gregory, 1980; Mumford, 1992; Friston, 2005; Pennartz, 2015). This constructivist view is supported, for instance, by the perceptual psychology of illusions (Gregory, 1980; Marcel, 1983) and by the uniform nature of action potentials conveying sensory information to the brain, unlabeled in terms of peripheral origin or modality (Pennartz, 2009, 2015). A promising computational paradigm for generating internal world models is predictive coding (Srinivasan et al., 1982; Dayan et al., 1995; Rao and Ballard, 1999; Lee and Mumford, 2003; Friston, 2005). Predictive coding models posit that higher areas of a sensory cortical hierarchy generate predictions about the causes of the sensory inputs they receive, and transmit these predictions via feedback projections to lower areas, which compute errors between predictions and actual sensory input. These errors are transmitted to higher areas via feedforward projections and are used for updating the inferential representations of causes and for learning by modifications of synaptic weights (Rao and Ballard, 1999; Bastos et al., 2012; Olcese et al., 2018).

In addition to being aligned with the feedforward/feedback architecture of sensory cortical hierarchies (Felleman and Van Essen, 1991; Markov et al., 2014), the occurrence of some form of predictive coding in the brain is supported by accumulating experimental evidence. Superficial layer V1 neurons in mice navigating in virtual reality code error signals when visual inputs are not matched by concurrent motor predictions (Keller et al., 2012; Leinweber et al., 2017; Keller and Mrsic-Flogel, 2018). Moreover, indications for a bottom-up/top-down loop structure with retinotopic matching were found by Marques et al. (2018) for a lower (V1) and higher (LM) area in mouse cortex. In monkeys, evidence for coding of predictions and errors has been reported for the face-processing area ML (Schwiedrzik and Freiwald, 2017). In humans, predictive coding is supported by reports of spatially occluded scene information in V1 (Smith and Muckli, 2010) and suppressed sensory responses to predictable stimuli along the visual hierarchy (Richter et al., 2018).

While foundational work has been done in the computational modeling of predictive coding, it is unknown how these early models – which were often hand-crafted and limited to only one or two processing layers (Rao and Ballard, 1999; Spratling, 2008, 2012; Wacongne et al., 2012) – can be expanded to larger and deeper networks in a way that can be considered neurobiologically plausible, or at least compatible with neurobiological principles. For instance, previous models studying attentional modulation or genesis of low-level response properties of V1 neurons (e.g., orientation selectivity) were limited to only a few units (Spratling, 2008) or to one processing layer devoid of top-down input (Spratling, 2010; Wacongne et al., 2012). These models provide a useful theoretical framework for studying information processing in early sensory areas but cannot be readily extrapolated to higher brain areas. A predictive coding approach for training deep neural networks was developed in Lotter et al. (2017) but this utilized the biologically implausible method of error-backpropagation for learning.

Thus we set out, first, to develop a class of predictive coding models guided by computational principles that allow architectures to be extended to many layers (i.e., hierarchically stacked brain areas) with essentially arbitrarily large numbers of neurons and synapses. Second, learning was required to be based on neurobiological principles, which led us to use unsupervised, gated Hebbian learning instead of physiologically implausible back-propagation based methods (Rumelhart et al., 1986; Lillicrap et al., 2016). The class of predictive coding models we introduce here is thus named “deep Hebbian predictive coding” (DHPC). Third, we investigated which properties associated with responses of biological neurons are also exhibited by model neurons without being explicitly imposed by network design constraints. For this purpose, we studied both low-level visual cortical properties such as orientation selectivity (Hubel and Wiesel, 1961) and high-level properties such as selectivity for whole images or objects found in, e.g., inferotemporal cortex (IT) (Gross et al., 1972; Desimone et al., 1984; Perrett et al., 1985).

## Materials and Methods

### Model Architecture With Receptive Fields

It is known that receptive field (RF) size increases from low to high-level areas in the ventral stream [V1, V2, V4, and IT] of the visual system (Kobatake and Tanaka, 1994). To incorporate this characteristic, neurons in the lowermost area of our network (e.g., V1) respond to a small region of visual space. Similarly, neurons in the next area [e.g., secondary visual cortex (V2)] are recurrently connected to a small number of neurons in V1 so that their small RFs jointly represent the larger RF of a V2 neuron. This architectural property is used in all areas of the network, resulting in a model with increasing RF size from lower-level to higher-level areas. Furthermore, there can be multiple neurons in each area having identical RFs (i.e., neurons that respond to the same region in visual space). This property is commonly associated with neurons within cortical microcolumns (Jones, 2000).

The model variants described in this paper receive natural images in RGB color model as sensory input of which the size is described by two dimensions representing the height and width of an image. Similarly, RFs of neurons in visual cortical areas extend horizontally as well as vertically. To simplify the explanation below, we will assume that the input to the network is one-dimensional and correspondingly neurons in the model also have RFs that can be expressed using a single dimension.

**FIGURE 1:**
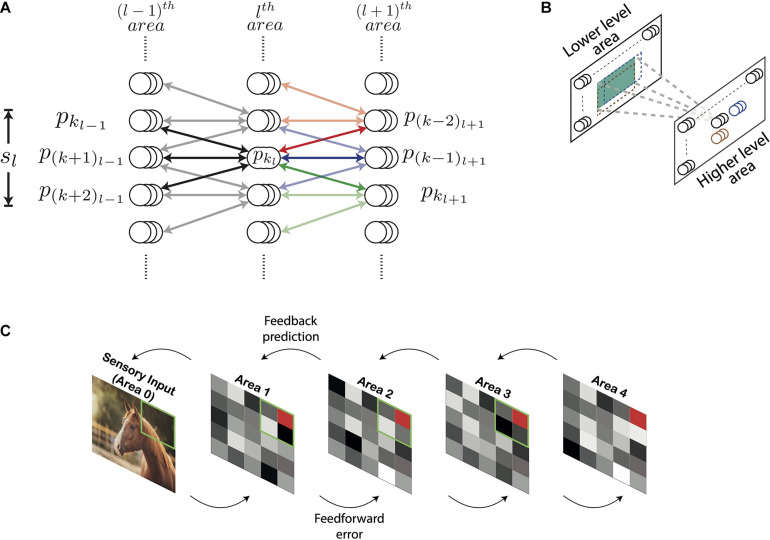
Architecture of the deep Hebbian predictive coding network with receptive fields. **(A)** A population of neurons having identical receptive fields is represented by three overlapping circles. *p*_*k_l_*_ denotes the *k*^th^ population in the *l*^th^ area and *s_l_* is the size of the receptive field of all populations in the *l*^th^ area. Both *s_l_* and *s*_*l*+1_ have been set to 3 here. For this value of *s_l_*, the populations *p*_*k*_*l*–1__ through *p*_(*k*+2)_*l*–1__ constitute the receptive field of the population *p*_*k_l_*_ (their connections are represented by black lines). Similarly, for this value of *s*_*l*+1_, *p*_*k_l_*_ will be present in the projective fields of populations *p*_(*k*–2)_l+1__ through *p*_*k*_*l*+1__. The populations within the receptive fields of *p*_(*k*–2)_*l*+1__, p(k-1)l+1 and *p*_*k*_*l*+1__ have been shown using red, blue, and green arrows, respectively. Their connections with *p*_*k_l_*_ are rendered in full color while other connections are shown in light colors. **(B)** For processing images, neuronal populations in each area can be visualized in a two-dimensional grid. Each population exhibits a two-dimensional receptive field (the receptive field of an example population in a higher-level area is shown in green). As a result, the receptive fields of two different populations can exhibit different overlaps horizontally and vertically. The receptive fields of two horizontally adjacent populations (black and blue) overlap completely in the vertical direction and partially in the horizontal direction. Similarly, the receptive fields of two vertically adjacent populations (black and brown) overlap completely in the horizontal direction and partially in the vertical direction. **(C)** An overview of the network with *n_l_* = 1 for all areas. Sensory input is presented to the network through Area 0. Activity of neurons in areas 1–4 is represented by tiles in grayscale colors. The green square in a lower area denotes the receptive field of the population represented as a red tile in the higher area.

Figure 1 shows the architecture of the DHPC network with (*N* + 1) layers which are numbered from 0 to *N*. The layers 1 to *N* in the network correspond to visual cortical areas; layer 1 represents the lowest area [e.g., primary visual cortex (V1)] and layer *N* the highest cortical area (e.g., area IT). Layer 0 presents sensory inputs to the network. Below, we will use the term “area” to refer to a distinct layer in the model in line with the correspondence highlighted above. Each area is recurrently connected to the area below it. Information propagating from a lower-level to a higher-level area constitutes feedforward flow of information (also termed bottom-up input) and feedback (also known as top-down input) comprises information propagating in the other direction. Conventionally, the term “receptive field” of a neuron describes a group of neurons that send afferent projections to this neuron. In other words, a RF characterizes the direction of connectivity between a group of neurons and a “reference” neuron. We employ a more general definition of RF in which the RF of a reference neuron in the *l*^th^ area is defined in terms of neurons in the (*l*−1)^th^ area. Specifically, the RF of a neuron *x* represents a group of neurons in a lower-level area that receive error signals based on predictions generated by higher-level neuron *x* (see section “Learning and Inference Rule”). Similarly, the group of cells that receive projections from a given neuron represents the projective field of that neuron. In the current paper the term “projective field” of a neuron *x* describes a group of higher-level neurons that receive error signals from the lower-level neuron *x* (see section “Learning and Inference Rule”).

Neurons in the *l*^th^ area are organized in populations of *n_l_* neurons having identical receptive and projective fields. Populations having an equal number of neurons are used to reduce computational overhead. The activity of the *k*^th^ population in the *l*^th^ area, referred to as *p*_*k_l_*_, is a (*n*_*l*_ by 1) vector denoted by **y**_*k*_*l*__. To reduce computational complexity, we assume that the RFs of all neurons in the *l*^th^ area are of equal size, denoted by *s_l_*, and the RFs of two consecutive populations have an overlap of (*s*_*l*_−1). The population *p*_*k_l_*_ is reciprocally connected with populations *p*_*k*_*l*–1__ through *p*_(*k*+*s_l_*−1)_*l*–1__ (Figure 1). Thus, the *l*^th^ area has (*s*_*l*_−1) fewer populations with distinct RFs compared to the (*l*−1)^th^ area. The synaptic strengths of connections between the populations *p*_*k_l_*_ and *p*_*k*_*l*–1__ is a (*n*_*l*−1_ by *n*_*l*_) matrix denoted by **W**_*k*_*l*−1_*k*_*l*__. We assume that the neuronal populations *p*_*k_l_*_ and *p*_*k*_*l*–1__ are connected by symmetric weights, i.e., feedforward and feedback projections between these populations have equal synaptic strengths. The top-down information transmitted by population *p*_*k_l_*_ to *p*_*k*_*l*–1__ is denoted by ${\hat{\text{y}}}_{k_{l-1}}^{{\text{k}}_{l}}$ and is given by

(1) $${\hat{\text{y}}}_{k_{l-1}}^{k_{l}}=\phi \left({\text{W}}_{k_{l-1}k_{l}}{\text{y}}_{k_{l}}\right)$$

where *ϕ* is the activation function of a neuron. Predictions (see section “Learning and Inference Rule”) about activities of the population *p*_*k*_*l*–1__ are denoted by ${\hat{\text{y}}}_{k_{l-1}}^{{\text{k}}_{l}}$. Neuronal activity is described in terms of firing rate, which by definition can never be negative. Therefore, we used a Rectified Linear Unit (ReLU) as an activation function which is defined as

(2) ϕ(x)=max⁡(x, 0)

which results in values that are positive or zero. To extend the architecture described above for handling natural images, the populations in each area can be visualized as a two-dimensional grid (Figure 1B). Here, each population has RFs that extend both horizontally as well as vertically.

### Learning and Inference Rule

The learning rule presented in this section is inspired by the approach to predictive coding in Rao and Ballard (1999) and builds upon our previous work (Dora et al., 2018). Each area of the model infers causes that are used to generate predictions about causes inferred at the level below. These predictions are sent by a higher-level area to a lower-level area via feedback connections. The lower-level area computes an error in the received predictions, as compared to its bottom-up input, and transmits this error to the higher-level area via feedforward pathways. The information received by an area is used to infer better causes, which is termed the *inference* step of predictive coding, and also to build the brain’s internal model of the external environment, which is termed the *learning* step.

**FIGURE 2:**
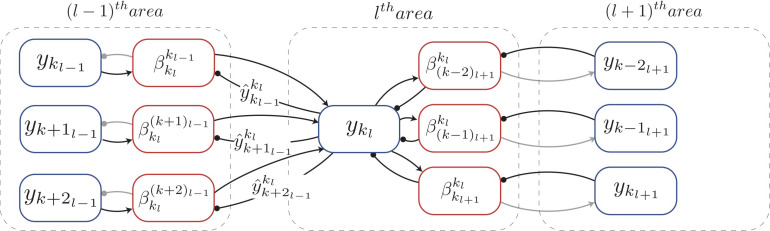
Biologically motivated realization of deep Hebbian predictive coding. Each rectangle denotes a population of neurons that represents a specific signal, computed in predictive coding. The populations that compute errors are denoted by red blocks and the populations that represent inferred causes are denoted by blue blocks. Arrows represent excitatory connections and filled circles denote inhibitory connections (note that inhibitory interneurons were not explicitly modeled here). The interareal connections between representation neurons and error neurons are plastic (Equation 8) whereas the intra-areal connections are not. Connections conveying information required for the inference and learning steps of predictive coding are shown as black lines and other connections are shown in gray. Gating mechanism has not been shown in this figure for simplification. See main text for explanation of symbols.

The neural implementation of predictive coding we developed is shown in Figure 2 for a one-dimensional sensory input. For a given sensory input, the neuronal activities ([**y**_1_*l*__, …, **y**_*k*_*l*__, …]) of all neurons in the *l*^th^ area collectively denote the causes of the sensory input inferred in this area, hence these neurons are referred as “representation neurons.” Based on these causes, the prediction of causes inferred in the (*l*−1)^th^ area is estimated according to Equation 1. Note that a given neuronal population in the *l*^th^ area will generate predictions only about the neuronal populations within its RF (Figure 2). This prediction in turn activates (when ${\hat{\text{y}}}_{k_{l-1}}^{{\text{k}}_{l}}$ is positive) or deactivates (when ${\hat{\text{y}}}_{k_{l-1}}^{{\text{k}}_{l}}$ is 0) a gating mechanism (not shown in Figure 2) which allows for inference and learning to occur (see below). Based on the prediction, the neuronal populations in the *l*^th^ area receive bottom-up errors via feedforward connections only from lower-level populations within their RF. Relative to area *l*, the bottom-up error $\left(\beta_{k_{l}}^{k_{l-1}}\right)$ based on the prediction generated by *p*_*k_l_*_ about the activity of *p*_*k*_*l*–1__ is computed as

(3) $$\beta_{k_{l}}^{k_{l-1}}=\left({\text{y}}_{k_{l-1}}-{\hat{\text{y}}}_{k_{l-1}}^{k_{l}}\right)$$

The computation of this bottom-up error occurs in the (*l*−1)^th^ area (red rectangles in Figure 2) and is transmitted to the *l*^th^ area via feedforward projections. The neurons in a given area that compute the bottom-up errors are termed “error neurons.” Note that the error neurons shown in Figure 2 were not included in Figure 1 for simplicity. The simulations in this paper use a summation of squared bottom-up errors $\left(e_{k_{l}}^{\beta }\right)$ received from populations in the RFs of *p*_*k_l_*_, which is given as

(4) $$e_{k_{l}}^{\beta }=\sum_{j=k}^{k+s_{l}-1}{\left(\beta_{k_{l}}^{j_{l-1}}\right)}^{2}$$

In general, other biologically plausible functions of bottom-up errors can also be used in simulations. Along with bottom-up errors, neurons in the *l*^th^ area also receive a top-down prediction from neurons in the (*l* + 1)^th^ area. Due to an overlap of (*s*_*l* + 1_−1) between two consecutive RFs in area (*l* + 1), populations in the *l*^th^ area will be present in the projective fields of *s*_*l*+1_ populations in the (*l* + 1)^th^ area (Figure 1A). Populations in the *l*^th^ area whose RFs are closer to the boundary of the visual space are an exception to this property as these neurons will be present in the projective fields of fewer than *s*_*l*+1_ populations. Here, we will focus on the general case. The population *p*_*k_l_*_ will receive top-down predictions from neuronal populations *p*_(*k*−*s*_*l*+1_+1)_*l*+1__ through *p*_*k*_*l*+1__. The error based on the top-down prediction of the neuronal activity of the population *p*_*k_l_*_ generated by the population *p*_*k*_*l*+1__ is computed as

(5) $$\beta_{k_{l+1}}^{k_{l}}=\left({\text{y}}_{k_{l}}-{\hat{\text{y}}}_{k_{l}}^{k_{l+1}}\right)$$

The computation of this top-down error occurs in the *l*^th^ area (Figure 2). In turn, this error will also constitute the bottom-up error for the population *p*_*k*_*l*+1__. Thus, whether an error signal is labeled bottom-up or top-down is defined relative to the area under scrutiny. The superscript and subscript in $\beta_{k_{l+1}}^{k_{l}}$ do not indicate a direction of signal propagation. The summation of squared errors due to the top-down predictions received by *p*_*k_l_*_ from *p*_(*k*−*s*_*l*+1_+1)_*l*+1__ through *p*_*k*_*l*+1__ is denoted by $e_{k_{l}}^{\tau }$ and is given as

(6) $$e_{k_{l}}^{\tau }=\eta \left(\sum_{i=k-s_{l+1}+1}^{k}{\left(\beta_{i_{l+1}}^{k_{l}}\right)}^{2}\right)$$

where η was set to one for all models unless specified otherwise (see section “Discussion”). In addition, we employ L1-regularization to counteract high levels of neuronal activity throughout all areas. Note that the regularization penalty is instated to suppress the average neuronal activity and does neither guarantee image selectivity nor representational sparseness in neuronal populations (see below). The neuronal activity of a given population is estimated by performing gradient descent on the sum of errors computed in Equations 4, 6, and L1-regularization on neuronal activities. This results in the following update rule for inferred causes

(7) $$\begin{array}{c} \Delta {\text{y}}_{k_{l}}=\\ -\gamma_{y}\left(\sum_{i=k-s_{l+1}+1}^{k}\beta_{i_{l+1}}^{k_{l}}-\sum_{j=k}^{k+s_{l}-1}g\left({\hat{\text{y}}}_{j_{l-1}}^{k_{l}}\right){\left(\beta_{k_{l}}^{j_{l-1}}\right)}^{T}{\text{W}}_{j_{l-1}k_{l}}+\alpha_{y}\right) \end{array}$$

where γ_*y*_ denotes the update rate for neuronal activities and α_*y*_ denotes the constant which controls how strongly the regularization penalty is imposed in comparison to other factors. The regularization penalty is equivalent to imposing a Laplacian prior on the estimated neuronal activities. Biologically, the Laplacian prior represents a passive decay of the activity of a population, at a rate determined by γ_*y*_ and α_*y*_ and irrespective of the stimulus and the current activation of the neuron. $g\left({\hat{\text{y}}}_{k_{l-1}}^{k_{l}}\right)$ in Equation 7 is a gating factor (equivalent to the partial derivative of the ReLU activation function), given by:

(8) $$g\left({\hat{\text{y}}}_{k_{l-1}}^{k_{l}}\right)=\left\{ \begin{array}{cc} 1 & \text{if}{\hat{\text{y}}}_{k_{l-1}}^{k_{l}}>0\\ 0 & \text{if}{\hat{\text{y}}}_{k_{l-1}}^{k_{l}}\geq 0 \end{array}\right.$$

Functionally, this operation is implemented by a gating mechanism which supports both inference and learning in the neural implementation of predictive coding (see below and section “Discussion”). All results presented here are based on a single model with a Laplacian prior. This is different from Rao and Ballard (1999) where Gaussian and sparse kurtosis priors were used for separate experiments. The update rule of Equation 7 constitutes the inference step of predictive coding. It results in causes that better match with top-down predictions and result in lower bottom-up errors. Higher-level areas thus influence the representations inferred in lower-level areas through top-down predictions. Similarly, lower-level areas affect the representations inferred in higher-level areas via bottom-up errors. To ensure that neuronal activities do not become negative after updating, we rectify the neuronal activities after every inference step using the rectifier function (Equation 2). Note that Δ**y**_*k*_*l*__ depends on the activities of neuronal populations that represent errors in the (*l*−1)^th^ and *l*^th^ areas and the synaptic strengths of the projections between populations in these two areas (Figure 2). All of this information is available locally to the population *p*_*k_l_*_.

The strengths of the synapses between populations in any two areas are updated using a (gated) Hebbian learning, resulting in gradient descent. Analogous to the inference step, an L1-regularization is imposed to avoid indiscriminately high values of synaptic strengths which imposes a Laplacian prior on the synaptic weights. Based on the errors defined in Equation 4 and L1 regularization of weights, the update rule for synaptic strength is given by:

(9) $$\Delta W_{k_{l-1}k_{l}}=-\gamma_{w}\left(-g\left({\hat{y}}_{k_{l-1}}^{k_{l}}\right)\beta_{k_{l}}^{k_{l-1}}{\left(y_{k_{l}}\right)}^{T}+\alpha_{w}\right)$$

where γ_*w*_ denotes the learning rate (governing synaptic weight changes) and α_*w*_ is the constant which determines how strongly regularization is imposed relative to other factors. The learning rule of Equation 9 constitutes the learning step of predictive coding. The term $g\left({\hat{\text{y}}}_{k_{l-1}}^{{\text{k}}_{l}}\right)$ in Equation 9 is not computed by a separate neural implementation but is functionally realized by the same gating mechanism as used in Equation 7. Consider the top-down prediction $\left({\hat{\text{y}}}_{k_{l-1}}^{{\text{k}}_{l}}\right)$ received by error neurons in the (*l*−1)^th^ area from the population *p*_*k_l_*_ (Figure 2). When, for instance, this prediction is 0, $\beta_{k_{l}}^{k_{l-1}}$ is equal to the neuronal activity (**y**_*k*_*l*−1__) of the population *p*_*k*_*l*–1__ (Equation 3). In this case, the update in the neuronal activity of the population *p*_*k*_*l*–1__ due to top-down error is proportional to the current activity of this population itself (Equation 7). Because $g\left({\hat{\text{y}}}_{k_{l-1}}^{{\text{k}}_{l}}\right)$ is 0, no modification occurs at the interareal synapse between the population *p*_*k_l_*_ and error neurons in the (*l*−1)^th^ area (Equation 9). In this case, the gating mechanism in the (*l*−1)^th^ area blocks flow of information onto the intra-areal synapse from representation neurons to error neurons, which prevents synaptic modification. When the prediction is positive, $g\left({\hat{\text{y}}}_{k_{l-1}}^{{\text{k}}_{l}}\right)$ is equal to 1, and therefore the interareal synapse between the population *p*_*k_l_*_ and the error neurons in the (*l*−1)^th^ area can be modified. Thus, the first term $\left(g\left({\hat{\text{y}}}_{k_{l-1}}^{{\text{k}}_{l}}\right)\beta_{k_{l}}^{k_{l-1}}{\left({\text{y}}_{k_{l}}\right)}^{T}\right)$ in Δ**W**_*k*_*l*−1_*k*_*l*__ is a (gated) Hebbian term as it depends on the activity of the population that represents bottom-up errors $\left(\beta_{k_{l}}^{k_{l-1}}\right)$ and the activity (**y**_*k*_*l*__) of *p*_*k_l_*_; these two are presynaptic and postsynaptic relative to each other, respectively (Figure 2). With “gated” we denote that plasticity is controlled by an additional factor controlling the amplitude of change, assuming a value of 0 or 1. The second term (α_*w*_) represents a Laplacian prior on the weights and is equal to the partial differentiation of the L1-norm of theweights being updated with respect to weights themselves. The (*n*_*l*−1_ *by n*_*l*_) matrix of synaptic strengths $\left({\text{W}}_{k_{l-1}}^{{\text{k}}_{l}}\right)$ between two populations can consist of both positive and negative weights. For a given weight (*w*) between the pre- and post-synaptic populations $\beta_{k_{l}}^{k_{l-1}}$ and **y**_*k*_*l*__, the second term equates to

(10) $$\alpha_{w}=\left\{ \begin{array}{cc} -1 & \text{if}w>0\\ 1 & \text{if}w<0 \end{array}\right.$$

which represents a passive decay of the weights. Based on this decay term, if w > 0, the weight will be updated to a value closer to 0, and the same holds for w < 0. The rate of this passive decay is determined by the product of the constants γ_*w*_ and α_*w*_. Thus, the learning rule in Equation 9 conforms to (gated) Hebbian plasticity. Note that it is used to update the synaptic strengths for interareal connections between error neurons and representation neurons whereas the intra-areal connections are not updated.

### Model Architecture Without Receptive Fields

In the generative model described in sections “Model Architecture With Receptive Fields” and “Learning and Inference Rule,” the representations in the *l*^th^ area of the model are optimized to generate an accurate prediction about causes inferred in the (*l*−1)^th^ area. In turn, this prediction about causes inferred in the (*l*−1)^th^ area can be used to generate a prediction about causes inferred in the (*l*−2)^th^ area. This process can be repeated until a prediction is generated about the sensory input in the lowest area. Using this method, it is possible to obtain a reconstruction of the sensory input using representations inferred in any area of the model. This functionality is shared with autoencoders (Hinton and Zemel, 1994). Note that information on the original sensory input is only coded by higher areas in the model by way of latent representations of the causes of sensory inputs. Here we use these reconstructions to qualitatively study the fidelity with which information about the sensory input is coded by the representations inferred in different areas. Our main goal is to study neural response properties in a cortex-like architecture with feedforward and feedback processing between areas, which deviates from the structure of autoencoders. Due to presence of overlapping RFs, neurons in each area generate multiple reconstructions of a single sensory input at the lowest level. This makes it harder to compare the reconstructions obtained using representations inferred in different areas of the model. To avert this problem, we built a network without RFs that is trained by the same method used for the network with RFs. In the network without RFs, each neuron in a given area is recurrently connected to each neuron in the areas below and above it. This fully connected network contained the same number of layers as the network with RFs and corresponding layers of the two networks contained equal numbers of neurons. A single reconstruction of each sensory input was obtained using the representations inferred in different areas of the network without RFs. Examples of these reconstructions are shown in the section “Model Without Receptive Fields: Inferred Causes Can Be Used to Reconstruct Sensory Input.” Besides the reconstructed sensory inputs, all other results reported here are based on the results obtained with the network having RFs.

### Details of Training

Both models are trained using 2000 images of airplanes and automobiles as sensory input and these were taken from the CIFAR-10 dataset. Each image has a height and width of 32 pixels. Table 1 shows the values of different hyperparameters associated with the architecture and learning rule. During training, stimuli were presented to the network in batches of 100. For each stimulus in a batch, the *inference* step (Equation 7) was executed 20 times in parallel in all areas and then the *learning* step (Equation 8) was executed once. Biologically, this corresponds to inferring representations of a sensory input on a faster time scale and updating the synapses of the underlying model on a longer time scale. At the beginning of training, the activity of all neurons in the network was initialized to 0.1 and the model was trained for 25,000 iterations.

**TABLE 1:** Hyperparameter settings used for training the network with and without receptive fields.

Hyperparameter	Meaning	Value (with RFs)	Value (without RFs)
*N*	Number of layers	4	4
*s*_*l*_, ∀*l* ∈ {1, 2, 3, 4}	Size of receptive fields	7	Fully connected
*n*_1_	Population size (Number of neurons in a population) in area 1	8	5408
*n*_*2*_	Population size in area 2	16	6400
*n*_*3*_	Population size in area 3	32	6272
*n*_*4*_	Population size in area 4	64	4096
γ_*y*_	Update rate for inference	0.05	0.0005
γ_*w*_	Learning rate for synapses	0.05	0.0005
α_*y*_	Regularization for causes	0.001 (all areas)	0.0001
α_*w*_	Regularization for weights	0.001 (all areas)	0.001

*The size of receptive field in the network with receptive fields is equal in both image dimensions. Note that the term receptive field (RF) has been used in this table in line with its conventional definition. For the network without RFs, n_1_, n_2_, n_3_, and n_4_ are equal to the total number of neurons in each area.*

Because the visual input image is of equal height and width, populations in areas 1–4 can be visualized in two-dimensional square grids. Areas 1–4 in the models presented here can be visualized using grids of sizes 26, 20, 14, and 8, respectively, which results in 676, 400, 196, and 64 populations in the respective areas. Each population in areas 1–4 contains 8, 16, 32, and 64 neurons, respectively, resulting in a total of 5408, 6400, 6272, and 4096 neurons (number of populations times population size), respectively. We varied several hyperparameter settings and observed that prediction errors started saturating when the ratio of the population size in a higher area to the population size in a lower area was higher than 2. In line with this observation, we trained models in which the population sizes were doubled in each successive area to ensure that lower predictions errors could be achieved across all model areas. Due to regularization and the rectification of causes after the inference step, some of the neurons remained inactive for any sensory input. These neurons were excluded from the analysis of activity patterns conducted in this paper, as they would not be detected by electrophysiological methods. At the end of a typical training session for a network with the neuron counts given above, 5393, 1280, 694, and 871 neurons were active in areas 1–4 of the network, respectively. The firing-rate responses of neurons across all areas in the model assumed values in the interval [0, 7.9].

To compute the number of synapses in the network, note that for every feedback synapse that transmits a prediction, there is a corresponding feedforward synapse that transmits an error (Figure 1). Thus, the number of feedforward and feedback synapses in the network is equal. The number of feedback synapses from a population (neurons with identical RFs) is equal to the product of the population size in higher-level and lower-level areas and the RF size in the higher level area. For example, the population size in areas 1 and 2 is 8 and 16 neurons (Table 1), respectively, and populations in area 2 have projective fields that extend by 7 units horizontally and vertically. This results in 6272 (7 × 7 × 8 × 16) feedback synapses from a given population in area 2. Thus, the total number of synapses between two areas is equal to 794,976 (area 0 and 1), 2,508,800 (area 1 and 2), 4,917,248 (area 2 and 3), and 6422528 (area 3 and 4; the number of populations times numbers of feedback synapses per population), respectively.

### Analysis of Neural Properties

Kurtosis is a statistical measure of the “tailedness” of a distribution. It is more sensitive to infrequent events in comparison to frequent events in the distribution. A commonly used definition of kurtosis, termed “excess kurtosis,” involves computing it for a given distribution with respect to the normal distribution. Under this definition, 3 (i.e., the kurtosis value of the normal distribution) is subtracted from the corresponding value of a given distribution. Given a set of observations (*x*_1_, …, *x*_*i*_, …, *x*_*N*_), excess kurtosis, henceforth referred to simply as kurtosis, is computed using the following equation:

(11) $$\kappa =\frac{\sum_{i=1}^{N}{\left(x_{i}-\bar{x}\right)}^{4}}{Ns^{4}}-3$$

where $\bar{x}$ and *s* denote the mean and standard deviation of the observations (*N* in total). Based upon the use of kurtosis as a measure of neuronal selectivity (Lehky et al., 2005) and sparseness (Lehky and Sereno, 2007) in experimental neuroscience, we employ it as a measure of these properties in our model. An estimate of kurtosis obtained from responses of a single neuron to all stimuli is used as an estimate of image selectivity. While computing selectivity, *N* will be equal to the number of stimuli. Similarly, its value obtained from the responses of all neurons to a single stimulus provides an estimate of sparseness. In this case, *N* will be equal to the number of neurons.

## Results

In this study we worked with two types of DHPC networks. The first type was a model without RFs, whereas the second model had RFs. Below we will first present results from the model without RFs. The aim of this first modeling effort was to examine if the network is well-behaved in the sense that latent representations of causes generated in higher areas can be effectively used to regenerate the sensory input patterns in lower areas, as originally evoked by input images. This regeneration was qualitatively evaluated as we did not set an explicit goal to achieve 100% accuracy. Following this section we will continue with DHPC networks with RFs, because this type of model is better suited to examine response properties of neurons across the respective areas along the visual processing hierarchy.

### Model Without Receptive Fields: Inferred Causes Can Be Used to Reconstruct Sensory Input

**FIGURE 3:**
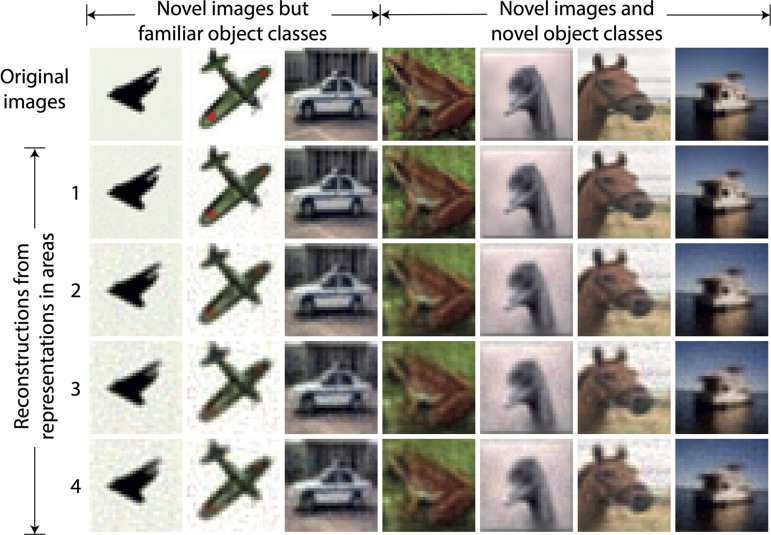
Examples of reconstructions obtained using causes inferred by the trained model without receptive fields. Each column represents an example of a sensory input. The three leftmost images represent novel stimuli from object classes used in training whereas other images are from object classes not used in training. The top row shows the novel sensory input that was presented to the network to allow it to construct latent representations across the areas. The second to fifth rows show the reconstructions of the sensory input obtained using the latent representations in the corresponding areas of the model. It can be observed that the reconstructed sensory input faithfully reproduces the novel originals, although the lower areas regenerate the inputs more sharply.

For the DHPC networks without RFs, we used a model that was trained on an image set *X* to infer causes for an image set *Y* that was never presented to the network during training. Set *X* contains images of objects from two classes, i.e., airplanes and automobiles, and set *Y* consists of images of 10 object classes namely airplanes, automobiles, birds, cats, deer, dogs, frogs, horses, ships, and trucks. Note that images of airplanes and automobiles in set *Y* were different from images of these object classes in set *X*. For a given stimulus in *Y*, a reconstruction of this stimulus is obtained using the causes inferred from each area of the model. For a given area, the inferred causes transmit a prediction along the feedback pathways to the level below. This process is repeated throughout the hierarchy until a predicted sensory input is obtained at the lowest level. Figure 3 shows examples of reconstructions of novel stimuli obtained using the causes inferred in each area of the model, along with the original sensory input. The first three exemplars are of airplanes and an automobile which belong to object classes that were used to train the model. The other exemplars are reconstructions of a frog, a bird, a horse, and a ship, which were never presented to the network during training, neither as exemplar nor as object class. We conclude that the reconstructions become somewhat blurrier if the generative process is initiated from higher, as opposed to lower, areas of the model, but also that the natural image statistics are captured reasonably well.

### Orientation Selectivity Emerges in a Lower Area of the Network With Receptive Fields

**FIGURE 4:**
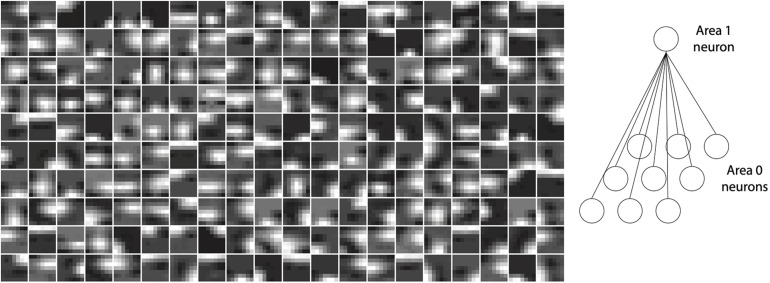
Orientation selectivity emerges in the lowermost area (area 1) of a trained model with receptive fields. Plots show normalized synaptic strengths for connections between area 1 and 0 (i.e., the input layer) of the model. Each box shows a symbolic representation of synaptic strengths from a randomly selected area 1 neuron to all area 0 neurons within its receptive field (right panel). Darker regions in the images correspond to normalized synaptic strengths closer to 0 and brighter regions in the images correspond to normalized strengths closer to 1. It can be observed that receptive fields of many cells contain non-isotropic patches imposing orientation selectivity on neural responses in area 1.

Neurons in V1 respond selectively to sensory input consisting of edges oriented at specific angles in their RFs (Hubel and Wiesel, 1961). The neurons in layer 1 of the model with RFs also exhibited this property. Importantly, this orientation selectivity was not hand-crafted or built into the network *a priori*, but emerged as a consequence of training the network on inputs conveying naturalistic image statistics. After training, the strengths of feedback synaptic connections between area 1 and 0 of the model resembled Gabor-like filters. Figure 4 plots the strengths of synapses onto a given neuron as representative examples for area 1 of the model (cf. Figure 1C). These plots were obtained by normalizing the feedback weights of a representation neuron in area 1 to the interval [0, 1]. Each image is obtained by rendering the normalized weights of a single representation neuron in area 1 as pixel intensities where each pixel corresponds to a specific neuron in area 0 in the RF of this representation neuron. Conventionally, orientation selectivity is viewed as a property of feedforward projections to V1. The model described here uses symmetric feedforward and feedback weights (apart from their difference in sign, Figure 2), therefore the orientation selectivity illustrated here is applicable to both feedforward and feedback connections between areas 0 and 1.

### Image Selectivity Increases Across Ascending Areas of the Model

Neurons in different brain areas situated along the sensory processing pathways exhibit tuning to features of increasing complexity. Whereas neurons in the primary visual cortex (V1) respond to edges of different orientations (see above) neurons in V4 respond selectively to, e.g., textures and colors (Okazawa et al., 2015) and neurons in IT show selectivity to particular faces or other objects (Gross et al., 1972; Tanaka et al., 1991; Perrett et al., 1992; Logothetis and Pauls, 1995). This property is manifested by differences in selectivity of cells across areas of the visual cortical hierarchy with later stages exhibiting higher selectivity in comparison to earlier stages. For our model, we asked whether analysis of area-wise neuronal activity would also reveal increasing selectivity from the lowest to highest areas.

**FIGURE 5:**
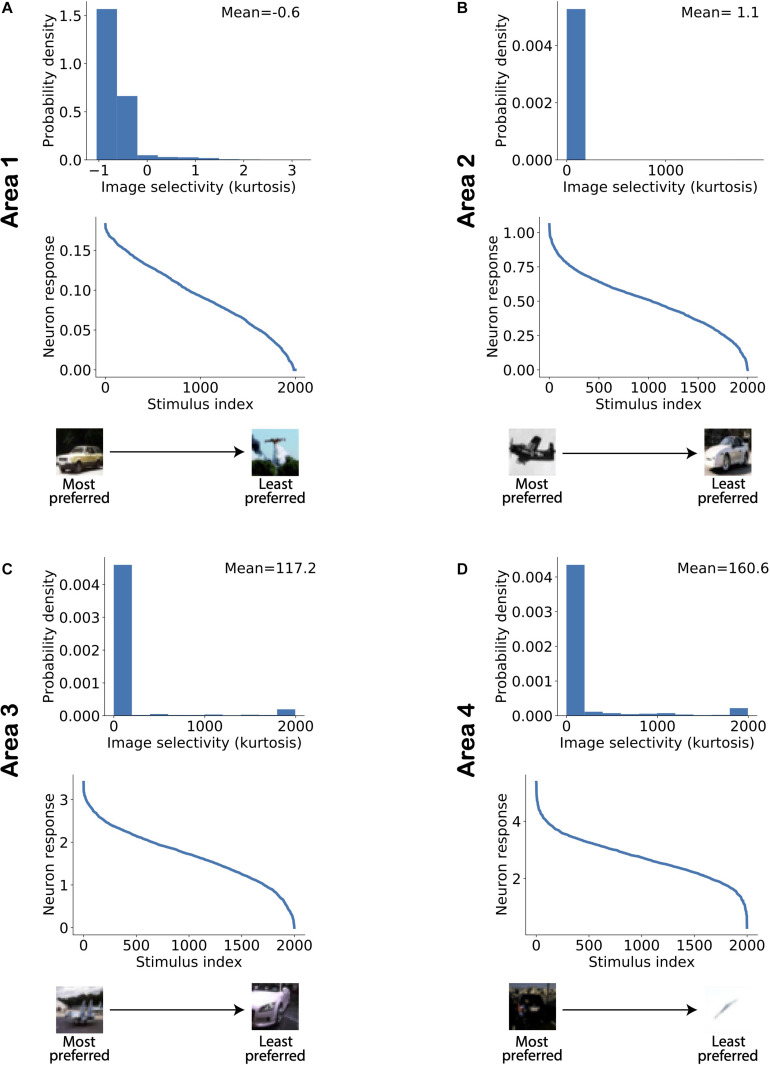
Image selectivity of model neurons increases across ascending areas of the model. **(A–D)** Distribution of image selectivity of neurons in each area of the model (top panels; **A:** lowest area/Area 1; **D:** highest area/Area 4). The mean value of neuronal image selectivity for each area is shown in the top right corner of the corresponding plots. (Bottom panel) The activity of a randomly chosen neuron in each corresponding area has been sorted according to its response strength for all stimuli presented to the network. It can be observed that the average selectivity of neurons increases from lower to higher areas in line with experimental data.

**FIGURE 6:**
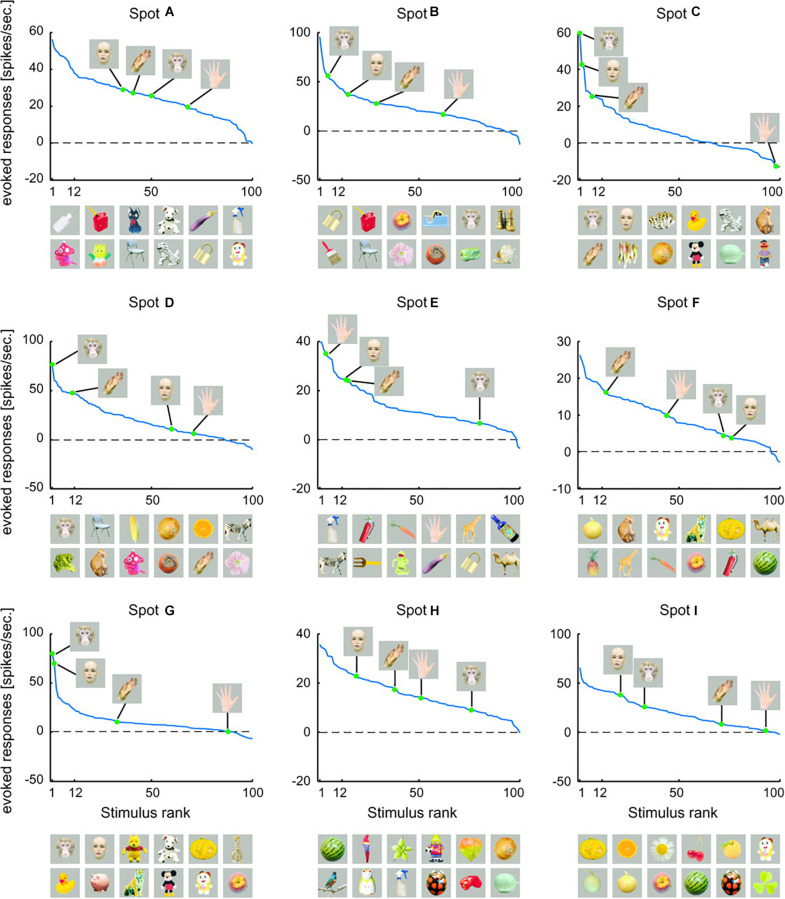
Rank-ordered responses to visual stimuli in monkey inferotemporal cortex. Firing-rate responses (spikes/s) to faces and hands of human and monkey recorded from different activity spots are plotted against stimulus rank. Here, activity spots refer to specific localized anatomical regions within inferotemporal cortex. The pictures below each figure represent the top 12 of preferred object stimuli, arranged in descending order from left to right. The upper row indicates the six most preferred images and the lower row indicates the 7th to the 12th best images [reproduced from Sato et al. (2009)].

Figure 5 shows the distribution of image selectivity for neurons in each area of the model. The kurtosis was computed for each neuron based on its responses to all stimuli presented to the model (Equation 10) and used as a measure of image selectivity for a single neuron (Lehky et al., 2005). The figure shows that neurons in all areas exhibit a strong response to a small number of images and that there are many images to which the neuron has a gradually weaker response. Similar response properties have also been reported in studies on areas along the visual processing hierarchy. Figure 6 shows example object tuning curves based on multi-unit recordings in monkey IT [reproduced from Sato et al. (2009); see also Suzuki et al., 2006]. Figure 5 shows that the mean image selectivity increases from the lowest to the highest area in the model. We compared the average selectivity in a given area with every other area in the model using Mann–Whitney’s *U*-test with Bonferroni correction for multiple comparisons. For all comparisons, the null hypothesis was rejected with *p* < 5.10^−15^. Thus, image selectivity strongly increased when ascending the model hierarchy.

### Sparseness Increases Across Ascending Areas of the Model

**FIGURE 7:**
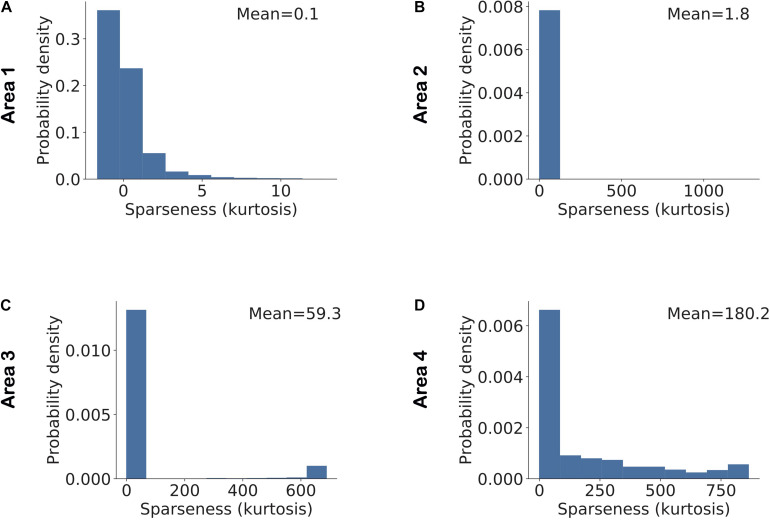
Sparseness in neuronal activity increases across ascending areas of the model. Sparseness was measured as the kurtosis across all neuronal responses in a given area and given a single stimulus. The mean value of sparseness is computed by averaging these estimates of kurtosis across all stimuli. **(A–D)** Distribution of sparseness in each area. The mean value of sparseness for each area is shown in the top right corner of each plot. It can be noted that the average sparseness of all neurons in model areas increases from lower to higher areas in agreement with some of the experimental studies.

A feature related to neuronal selectivity is sparseness, reflecting how scarcely or redundantly a feature or object is coded across the population in a given area (Vinje and Gallant, 2000; Willmore and Tolhurst, 2001; Perez-Orive et al., 2002; Montijn et al., 2015). A high or low sparseness can easily arise in a population with large variations in average cellular activity. For instance, consider a population in which a single neuron has an average firing rate of 100 spikes/s and all other neurons have an average firing rate of 10 spikes/s. In this population, the peak in the distribution of population activity due to the neuron with high average activity will result in high sparseness. To overcome this problem in the analysis, we normalized the activity of all model neurons using their average activity and an individual estimate of kurtosis was obtained for each stimulus across all neurons in each area based on this normalized activity. Figure 7 shows a distribution of sparseness in each area. We found that the average value of sparseness across all stimuli in each area increased systematically from the lowest to highest area. For validation, we conducted a pairwise comparison of sparseness values in different areas using Mann–Whitney’s *U*-test with Bonferroni correction for multiple comparisons. For all comparisons between areas, the null hypothesis was rejected with *p* < 5.10^−34^.

We found that these results were strongly dependent on regularization in the network. In the absence of any regularization, average sparseness first increased and then decreased when ascending across areas (Supplementary Figure 1). This can be attributed to the network property that all areas in the model infer causes that reconcile bottom-up and top-down information (Equations 4, 6) received by an area, except for the top area where causes are determined only by bottom-up information. This lower constraint on the top area leads to a decrease in sparseness in areas farther away from the sensory input layer. Imposing regularization only on representations inferred in the top area to compensate for this lack of constraint did not alter this pattern of average sparseness across model areas (Supplementary Figure 2). Further analysis showed that this phenomenon occurred because sparse neuronal activity in higher areas induced by regularization results in sparse top-down predictions to lower areas which indirectly induce sparseness in representations inferred in lower areas. Thus, average sparseness in areas is determined by multiple factors pertaining to learning and inference. Differences in these factors across experimental studies may help explain why previous experimental studies on visual cortex have reported diverging results on sparseness (see section “Discussion”).

Furthermore, high regularization led to neurons being active for only a small number of images. When the activity of such neurons was normalized by their mean activity, this could result in very high (relative) activity for some of these images. An estimate of kurtosis obtained from normalized neuronal activity can thus lead to arbitrarily high estimates of sparseness (Figure 7).

### Selectivity Is Negatively Correlated While Sparseness Is Weakly Correlated With the Average Neuronal Response

We next studied the relationship between a neuron’s selectivity and its average response to all stimuli. Similarly, for each area of the model we also investigated the relationship between the average response of all neurons in an area to a stimulus and the sparseness estimate for that area. The selectivity in different areas of the model exhibited wide variations. For the purpose of visualizing how the relationship between selectivity and mean neuronal activity evolves from lower to higher areas, we looked at the relationship between the log of selectivity and mean neuronal activity. We observed that, in all areas, there was a negative correlation between the selectivity and average neuronal activity, i.e., neurons with high selectivity had low average activity. Pearson correlation coefficients of −0.23, −0.05, −0.55, and −0.42 were obtained between selectivity and mean responses in areas 1–4, respectively. This has also been reported in experimental data (Lehky et al., 2011). Further, this negative correlation became stronger from lower to higher areas in the model.

We conducted a similar study on the relationship between sparseness and average population activity. It has been reported in experimental data that the average population response shows little variation for different values of sparseness (Lehky et al., 2011). This was also the case for all model areas as we observed only weak correlations between sparseness and average population responses. Pearson correlation coefficients of −0.18, 0.02, 0.23, and 0.18 were obtained between sparseness and mean responses in areas 1–4, respectively. These similarities between the statistical properties of model neurons and data from animal experiments arise without being imposed by a targeted network design or training procedure. The weak correlations between sparseness and average firing rate of all neurons in a given area imply that the responses of neurons in that area to different stimuli vary in terms of their distributed activity pattern, while the average firing rate across all neurons in the area does not change significantly for various stimuli. This behavior was observed for all areas in the model. Functionally, this may enable a sensory cortical system to keep the average firing rate in an area relatively constant across stimuli, while exhibiting distinct activity patterns to these stimuli, which is useful for stimulus discrimination capacities and efficient energy consumption.

### Regularization Determines Whether Sparseness Depends on Highly Selective Neurons or Neurons With High Dynamic Ranges

Although selectivity and sparseness represent different aspects of neuronal activity, they are interconnected quantities, i.e., a population consisting of highly selective neurons will also exhibit sparseness in the population response to a single stimulus. However, data recorded from macaque IT show that the dynamic range of single-cell responses correlates more strongly with sparseness than selectivity (Lehky et al., 2011). Here, dynamic range was quantified using the interquartile range of neuronal responses, which is the difference between the 75th and 25th percentiles of a neuron’s responses to the individual stimuli presented. We asked which of the two factors, selectivity or dynamic range, contributed to sparseness in the responses of model neurons in different areas.

**FIGURE 8:**
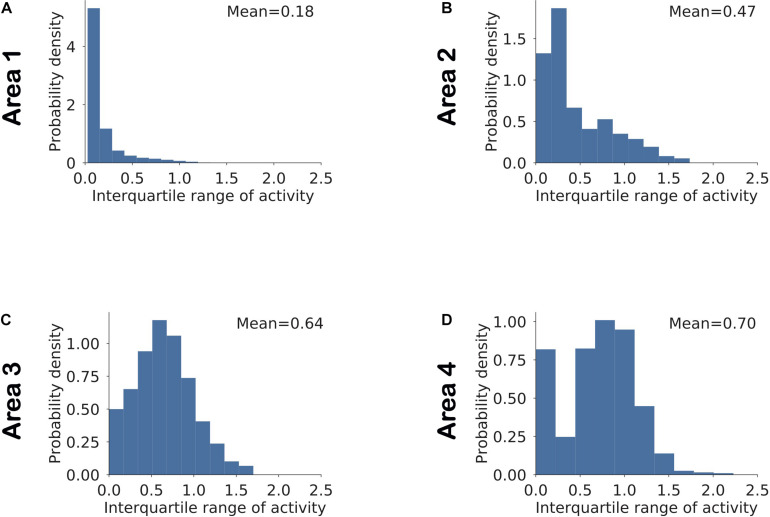
**(A–D)** Distribution of the dynamic range of neurons computed as the interquartile range of the neuronal responses in a given area across all stimuli. The mean value for each area is computed by averaging across interquartile ranges for all neurons in that area.

**FIGURE 9:**
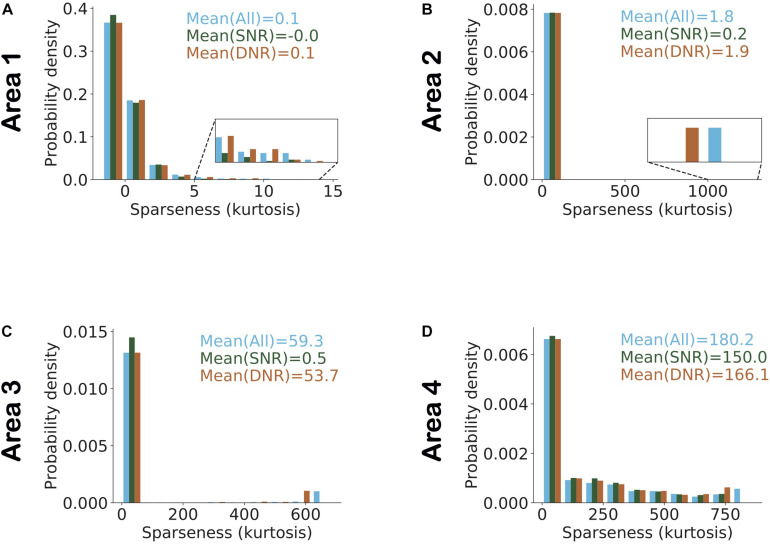
Highly selective neurons determine sparseness more strongly in comparison to neurons with high dynamic range. **(A–D)** Histogram of sparseness for three different populations of neurons. The distribution of sparseness was first determined with all neurons in an area included, and is shown in blue. The population in which the top 10% most image-selective neurons were removed (SNR) is shown in dark green and light brown denotes the populations in which the top 10% neurons with high dynamic response range were removed (DNR). The top 10% selective neurons that were removed here, were identified based on their image selectivity (cf. Figure 5). Neurons in the top 10% of the dynamic range that were removed were identified based on their interquartile ranges (cf. Figure 8). Values represent the mean sparseness estimates for the different populations in corresponding colors. In all areas of the model (except area 1) it can be observed that the mean sparseness drops much more strongly on removal of highly image-selective neurons in comparison to removal of neurons with high dynamic range.

To examine the interactions between these network parameters, we estimated sparseness in three different sets of neuronal populations that differed in terms of selectivity and dynamic range. Figure 8 shows the histogram of interquartile ranges for neurons in each area. The dynamic range gradually increased from lower to higher areas as more neurons shifted away from low range values. For each area, we considered a first subset, denoted by “SNR” (i.e., Selective Neurons Removed), obtained by removing activities of the top 10% of neurons having the highest selectivity in that area (Figure 8). To obtain the second subset of each area, denoted by “DNR” (i.e., Dynamic Range Neurons Removed), we eliminated the activities of the top 10% of neurons with the broadest interquartile ranges. Figure 9 also shows the distribution of sparseness of the third set, viz., including all neurons of an area (denoted by “All”). It can be clearly seen that sparseness is more dependent on neurons with high selectivity in comparison to neurons that exhibit a broad dynamic range. Thus, our model shows a strong influence of neuronal selectivity on sparseness. This model behavior was also dependent on regularization.

In the absence of regularization, sparseness in lower areas was determined by high selectivity neurons, but in higher areas sparseness was determined by high dynamic range neurons (Supplementary Figure 3). This can be attributed to the network property that the bottom-up input to lower areas is more strongly driven by a fixed sensory input whereas in higher areas the bottom-up drive is based on constantly evolving representations. Stochastic fluctuations resulting from these evolving representations at the inference step in higher areas lead to higher dynamic response ranges in these very areas. As a result, sparseness is more strongly determined by high dynamic response range neurons in higher areas, which is in line with the experimental results of Lehky et al. (2011). However, adding regularization to the top area constrains neural activity in higher areas, thereby reducing the dependence of sparseness on high dynamic range neurons (Supplementary Figure 4).

### Area 4 Exhibits Higher Object Classification Performance Compared to Lower Model Areas

**FIGURE 10:**
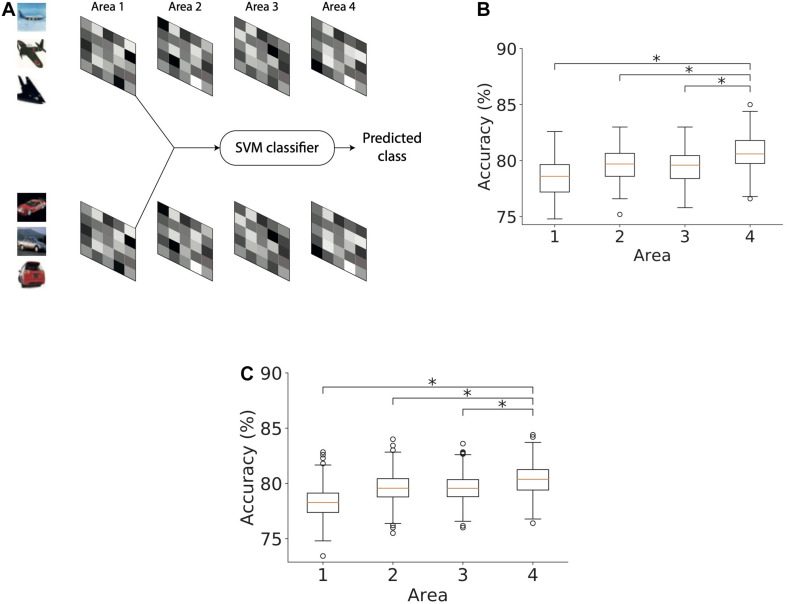
Object classification performance based on Area 4 representations is higher than that based on lower model areas. **(A)** Method used for computing the accuracy of a classifier based on causes, in this case, inferred in area 1. The inferred causes for a given stimulus are presented to a support vector machine (SVM) classifier whose output is used to determine the predicted class (airplanes versus cars) of a given stimulus. This procedure is repeated for all areas. **(B)** Boxplot of classification performance in different areas using 1500 randomly selected samples for optimization. Horizontal lines of the boxes denote the first, second, and third quartiles. Whiskers represent the entire range of data and circles denote outliers. The second quartile in all areas was significantly above chance level accuracy (one sample *t*-test, *p < 0.05). The performance of the classifier optimized using area 4 representations was significantly higher than the performance of classifiers of other areas (Mann–Whitney’s *U*-test with Bonferroni correction, *p < 0.05). **(C)** Boxplot of classification performance in different areas using different numbers of samples for optimization. The number of samples did not affect the conclusion observed in panel **(B)** (Mann–Whitney’s *U*-test with Bonferroni correction, *p < 0.05).

We next studied the ability of the model with RFs to infer causes that generalize across different exemplars of a given object class. The exemplars varied in terms of object identity, viewing angle, size, etc. For this purpose, we trained separate support vector machine (SVM) classifiers using latent representations of causes in each of the four areas of the model (Figure 10A). We split the set of images using a 75–25 ratio where 75% of the images were used for training and 25% of them were used for evaluating the classification performance. Using the training subset of the stimuli with which the model was trained, a linear binary SVM classifier was optimized to distinguish between representations of exemplars of two object classes, i.e., airplanes and automobiles. The remaining stimuli (25% of the images) were used to estimate the performance of the SVM classifier which thus yields an estimate of the model’s capacity to generalize across different exemplars of the same class. The percentage of latent representations that was correctly categorized by the trained binary SVM classifier was used as an estimate of object classification performance.

To examine whether the representations in different areas exhibited better generalization progressively across ascending areas, we optimized a linear SVM classifier using representations for 1500 stimuli randomly chosen from both classes and then computed its classification performance on the remaining 500 stimuli. This analysis was repeated 100 times by bootstrapping without replacing the samples selected for optimizing the linear SVM classifier. Figure 10B shows the classification performance of the SVM classifier for representations in different areas of the model. First, we observed a classification accuracy well above chance level in all areas (one sample *t*-test; *p*-values are lower than 8.10^−130^ for all areas). Second, we observed a modest but systematic increase in the classification performance from the lowest to highest area of the model. This shows that representations in higher areas can generalize better across unfamiliar exemplars than lower areas. To validate our results, we compared the accuracy in the topmost area with accuracy in other areas using Mann–Whitney’s *U*-test with Bonferroni correction for multiple comparisons. The maximum *p*-value of 0.0004 was obtained for the comparison between the accuracies of the topmost area and area 2. Based on these comparisons, the null hypothesis for all comparisons between areas was rejected at a significance level of at least 0.01.

To ensure that this result was not dependent on the number of stimuli used, we repeated this analysis with different stimulus sets. For this purpose, we optimized the SVM classifier on stimulus sets containing 1000–1500 stimuli in steps of 100 and evaluated its performance on the remaining stimuli. Figure 10C shows the performance of the classifiers optimized using different numbers of stimuli for different areas of the model. The generalizing capacity of the inferential representations in higher areas of the model was better than in the lower areas irrespective of the number of stimuli used to optimize the SVM classifier. For all comparisons, the null hypothesis could be rejected at a significance level of at least 0.05. The lowest level of significance was obtained for the comparison between the accuracies of the top area and area 2 (*p* < 1.10^−21^).

## Discussion

We described a general method to build deep predictive coding models for estimating representations of causes of sensory information, based on principles compatible with neurobiology. Different hyperparameters of the network can be modified to model various aspects of cortical sensory hierarchies; for instance, *N* was varied from 1 to 5 to study cortical hierarchies of increasing depth. This provides a mechanism to develop deep neural network models that can be used to simultaneously study properties of lower-level as well as higher-level brain areas. The models were trained using unsupervised (gated) Hebbian learning. Both the inference and learning steps utilized only locally available information. We found that several properties of neuronal and population responses emerge without being imposed *a priori* by network design or by the inference and learning steps. Image selectivity increased systematically from lower to higher levels, even in a linear model with no regularization of weights and representations, and the average sparseness of representations increased from lower levels to higher levels, which has been reported in experimental work (Okazawa et al., 2017).

Furthermore, we studied object classification properties of the causes inferred by the model. The classifiers optimized using representations in higher areas exhibited better performance in comparison to those in lower areas. Thus, predictive coding may provide a useful basis for the formation of semantic concepts of increasing complexity along the information processing hierarchy in the brain, at least when combined with networks performing categorization [e.g., in the medial temporal lobe (Quiroga et al., 2005) or prefrontal cortex (Freedman et al., 2003)].

### Reproduction of Experimental Findings by the Model

The increase in image selectivity in ascending areas of DHPC networks has also been reported in experimental studies on visual cortical areas (Gross et al., 1972; Tanaka et al., 1991; Logothetis and Pauls, 1995). This can be attributed to the strong activation of neurons in each model area by the patterned activity of neurons within their RF. For example, neurons in the lowest area develop Gabor-like filters that resemble oriented edges which have also been shown to emerge naturally in theoretical models based on efficient coding of sensory input (Barlow, 1961; Olshausen and Field, 1996; Chalk et al., 2018). These low-level neurons will be strongly active when a particularly oriented edge is present within their RF. Similarly, a neuron at the next level will be strongly active when neurons within its RF at the lower level exhibit a specific pattern of activity. A neuron at this higher level will therefore only become active when a particular configuration of edges (rather than a single edge) occurs at a specific location in visual space, resulting in an increase in complexity of features coded at this level. This increased complexity in successive model areas leads to a corresponding increase in the average neuronal selectivity when ascending the hierarchy.

It could be argued that regularization will automatically lead to an increase in average selectivity in neuronal responses across model areas. To examine this possibility, we also trained linear models without regularization (either for synaptic weights or inferred causes) while all other hyperparameters remained unchanged. These models also exhibited an increase in average selectivity across model areas, underscoring the conclusion that this increasing selectivity is an emergent network property, not solely imposed by regularization. However, adding regularization did result in an overall increase in average selectivity in each model area. By definition, the responses of a selective neuron will have a high interquartile range. Thus, the increasing selectivity across model areas also leads to an increase in the average interquartile range across ascending model areas (Figure 8).

Unlike selectivity, there is no consensus in the literature on how sparseness varies along the cortical hierarchy due to a lack of consistency in experimental data. Experimental studies indicate either an increase (Okazawa et al., 2017) or constancy of sparseness along the cortical hierarchy (Rust and DiCarlo, 2012). We observed that variations in average sparseness across model areas depended strongly on multiple factors which include the hierarchical position of an area, the regularization and the difference in weighting of bottom-up versus top-down feedback. A lack of top-down feedback in the top area resulted in lower average sparseness in areas closer to the top compared to lower areas. Thus, a low average sparseness at the top spreads to other areas in the network (Supplementary Figure 2). Similarly, having regularization in the top area leads to increased sparseness in other areas, with areas closer to the top exhibiting a stronger increase in sparseness (Supplementary Figure 2). Having regularization at the top significantly reduced the difference in sparseness between areas 1 and 4 (Supplementary Figure 2), which aligns with experimental observations reported by Rust and DiCarlo (2012). These effects could be altered by changing the relative strength assigned to bottom-up and top-down feedback. For instance, in a model with regularization in the top area and η < 1 (Equation 6), the average sparseness increased from lower to higher areas as observed in Okazawa et al. (2017). These results may help explain the varying results regarding sparseness observed in experimental data. Thus, different settings associated with the three factors that impact sparseness (viz., hierarchical position of an area, regularization and difference in weighting of bottom-up versus top-down feedback) may support various sparseness regimes across the information processing hierarchy, thereby enabling exploration of dynamic coding behaviors in the brain. In experiments, sparseness has been compared across two brain regions at most, and our model suggests that results obtained from such studies may not generalize to other brain regions.

Regularization also affected the contributions of high-selectivity neurons or high-dynamic range neurons to sparseness (Figure 9). Having regularization in an area suppressed the average neural activity in this area, thereby reducing the dependence of sparseness on high dynamic range neurons (Supplementary Figure 4).

### Object Classification Performance

We showed that a binary SVM classifier optimized using higher-level representations (causes inferred in area 4) performed better than a classifier trained on lower-level representations (i.e., in areas 1, 2, and 3). This effect disappeared when there was no regularization penalty. Regularization of activity and synaptic strength biased the network to generate representations in which most neurons were inactive (or less active) and active neurons captured most of the information in the presented stimuli. This results in a representational code that allows better discrimination between object classes. Thus, regularization helps improve the accuracy of the classifiers based on representations in each area significantly above chance level. In combination with increasing feature complexity in the network, this leads to a modest but systematic increase in classification performance from lower to higher levels in the network.

### Comparison With Previous Models

Existing works on predictive coding models have provided a solid foundation for studying various properties of neuronal responses in early sensory areas (Rao and Ballard, 1999; Spratling, 2008, 2010, 2012). For instance, it has been shown that a predictive coding network with two cortical regions and suitable initialization of synaptic strengths can reproduce various aspects related to attention (Spratling, 2008). An extension of this model reproduced various properties associated with neuronal responses in V1 (Spratling, 2010). A different model of predictive coding that employed neurons selective to different auditory tones arranged in a columnar architecture accounted for mismatch negativity (Wacongne et al., 2012). DHPC networks advance upon these studies by providing a methodology for building scalable, deep neural network models using a (gated) Hebbian rule for both adjusting synaptic strengths and estimating inferential representations. It can be used as a framework to study more complex aspects of information processing that rely on higher level areas in the brain.

Deep Hebbian predictive coding networks provide a mechanistic framework for predictive processing with arbitrary and scalable architectural attributes corresponding to biological analogs like RF size and number of brain areas. Here, DHPC networks were scaled up to contain millions of synapses and thousands of neurons whereas most existing predictive coding models have simulated networks with up to hundreds of neurons and thousands of synapses. Furthermore, DHPC networks reproduce, within the same architecture, many attributes of neuronal responses without explicit *a priori* incorporation of these properties in the model. Probably, the approach closest to our work is by Lotter et al. (2017) who employed networks consisting of stacked modules. This network was specifically designed to predict the next frame in videos and was trained end-to-end using error-backpropagation, which is unlikely to be realized in the brain.

### Neurobiological Plausibility and Anatomical Substrate of Predictive Coding

Deep Hebbian predictive coding networks employ a learning rule (Equation 9) that consists of a Hebbian term depending on the activity of pre- and post-synaptic neurons, a gating factor, and an additional passive decay term. The decay term leads to a passive decrement of established weights toward zero and is determined by the learning rate (γ_*w*_) and the factor (α_*w*_) that determines the strength of the regularization penalty. As concerns the gating factor, these networks do not compute the derivative of the ReLU activation function explicitly, instead they deploy a gating mechanism to realize well-behaved learning and inference. There are multiple possibilities for implementing this gating mechanism neurobiologically, such as neural circuits modulating presynaptic activity [for instance, modulation of transmitter release via metabotropic glutamate receptors (Takahashi et al., 1996)], effects of neuromodulators [for instance, presynaptic regulation of glutamate release by nicotinic acetylcholine receptors (McGehee et al., 1995; Gray et al., 1996)] or synapse- or dendritic compartment-specific postsynaptic modulation such as by somatostatin-positive cortical interneurons (Yaeger et al., 2019) or norepinephrine (Lur and Higley, 2015).

Importantly, the learning rule of Equation 8 is only employed for modifying the synaptic strengths of interareal connections between lower-level error neurons and higher-level representation neurons. Intra-areal connections between representation neurons and error neurons are not modified (Figure 2). This restriction might seem biologically implausible at first sight, but previously it has been emphasized that the brain requires mechanisms for controlling plasticity to preserve previously acquired knowledge while maintaining the capability to continue learning from new experiences (McClelland et al., 1995). GABAergic inhibition has been suggested as a means for controlling plasticity in the brain (Wigström and Gustafsson, 1986; Pennartz et al., 1993; Wilmes et al., 2016), while simultaneously permitting transmission of information in the presence of strong excitation. Although we did not incorporate these inhibitory mechanisms explicitly in our model, our results illustrate how localized inhibition in representation and error neurons may usefully allow for plasticity of interareal synapses while suppressing modification of intra-areal synapses in DHPC networks. Inhibition of representation neurons could specifically suppress plasticity induced by information transmitted over synapses from the intra-areal error neurons. Similarly, inhibition of error neurons could suppress synaptic modification induced by information transmitted over synapses from the intra-areal representation neurons. Thus, a biological realization of DHPC networks in the brain may rely on existence of localized GABAergic inhibition between intra-areal representation and error neurons (or another mechanism to prevent plastic changes of intra-areal connections, such as a lack of NMDA receptors) instead of a network with homogeneous connectivity between intra-areal neurons (for example, Garagnani et al., 2008).

As concerns the regularization penalty on high neural activity (Equation 7), this may be biologically realized through multiple mechanisms such as, again, GABAergic inhibition [for example by inhibition of pyramidal cells through parvalbumin-positive interneurons (Perrenoud et al., 2016; Tremblay et al., 2016)], normal repolarization of the neuron toward resting membrane potential following perturbation, or spike frequency adaptation [for example through Calcium-dependent Potassium currents (Khawaja et al., 2007)].

An intriguing question related to predictive coding is its potential neuroanatomical substrate in the brain. Several studies have looked at possible biological realizations of predictive coding based on physiological and anatomical evidence (Bastos et al., 2012; Keller and Mrsic-Flogel, 2018; Pennartz et al., 2019). DHPC networks are well compatible with insights from several experimental studies on predictive coding and error signaling (Leinweber et al., 2017; Schwiedrzik and Freiwald, 2017) and cortical connectivity (Rockland and Pandya, 1979; Douglas and Martin, 2004; Marques et al., 2018). However, some aspects of predictive coding highlighted by experimental studies have not yet been explicitly modeled by the current DHPC architecture. A combination of experimental and modeling studies predicts that neurons coding inferential representations are present in superficial as well as deep layers of sensory cortical areas (Pennartz et al., 2019). Representation neurons in deep layers are proposed to transmit top-down predictions to error neurons located in superficial layers of the lower area they project to (Bastos et al., 2012; Pennartz et al., 2019). These error neurons also receive input from local representation neurons in superficial layers of the same area and transmit bottom-up errors to the granular layer of the higher area they project to.

This anatomical configuration and the neurophysiological differences in neuronal properties across neocortical laminae are not considered in the current architecture. This would require explicitly modeling various cell types located in different neocortical layers to study the impact of different activation properties of the various cell types on network properties. For simplicity, our DHPC networks employ a ReLU activation function across all layers and we have therefore assumed that the neurons are operating in a bounded range (i.e., their activity lies between zero and the upper bound of the near-linear shape of a sigmoid activation function). The firing rates of representation neurons in the model are arbitrary and, using an appropriate scaling factor, could be mapped to firing rates observed in the cortex. Further, the proposed DHPC networks utilize a simple layered network in which areas are reciprocally connected with their immediate neighbors. This architecture does not take into account the existence of long-range connections in the brain, for instance, direct occipito-temporal connections (Gilbert and Li, 2013; see also Pennartz et al., 2019 for “skip connections”).

Another simplifying assumption made by DHPC networks is the existence of bi-directional, interareal connections with the same synaptic strength between representation neurons in a higher area and error neurons in a lower area. Further, feedforward and feedback connectivity in DHPC networks is configured such that the RFs of lower-level neurons and those of higher-level neurons that predict activities of these lower-level neurons overlap with each other. In mice, feedback from a higher visual area (i.e., lateromedial cortex, LM, to V1) targets retinotopically matched locations, which supports the assumption of overlapping RFs for lower- and higher-level neurons (Marques et al., 2018). As yet, there is no evidence on correlations between synaptic strengths of feedforward and feedback connections between higher visual areas and V1. However, randomly initialized feedforward and feedback connections between representation and error neurons may well become correlated when updated using Hebbian mechanisms. This may be attributed to the fact that update rules for both feedforward and feedback connections rely on the same set of correlated pre- and post-synaptic activities. A Hebbian update rule has been shown to be effective in training deep neural networks with non-symmetric feedforward and feedback connections (Amit, 2019). Another possibility to address this neurobiological question is the theory of feedback alignment (Lillicrap et al., 2016) which suggests that modifiable feedforward weights may adapt to the information transmitted by randomly initialized feedback weights, thereby alleviating the need for an *a priori* constraint on symmetrical weights.

Altogether, these considerations reveal a number of constraints that are required to allow for well-behaved learning and inference in deep predictive coding networks operating on a Hebbian basis, and which are compatible with neurobiological principles identified in cortical architectures. Before considering this class of models as neurobiologically plausible, however, more tests will have to be conducted, guided by the various predictions derived from the DHPC inference and learning steps. As such, they may inform future research and will help bridge the gap between theoretical models and biologically relevant aspects of cortical architectures potentially implementing predictive coding.

## Data Availability Statement

The raw data supporting the conclusions of this article will be made available by the authors, without undue reservation.

## Author Contributions

SD, SB, and CP conceived the study and developed the framework. SD wrote the code for architecture, training, and analysis. SD and CP revised the text and figures. All authors contributed to writing the article and approved the submitted version.

## Conflict of Interest

The authors declare that the research was conducted in the absence of any commercial or financial relationships that could be construed as a potential conflict of interest.

## Publisher’s Note

All claims expressed in this article are solely those of the authors and do not necessarily represent those of their affiliated organizations, or those of the publisher, the editors and the reviewers. Any product that may be evaluated in this article, or claim that may be made by its manufacturer, is not guaranteed or endorsed by the publisher.
