# Supplementary material for: Deep Gated Hebbian Predictive Coding Accounts for Emergence of Complex Neural Response Properties Along the Visual Cortical Hierarchy
Source: Front Comput Neurosci. 2021 Jul 28;15:666131. doi: 10.3389/fncom.2021.666131 (PMC8355371; doi:10.3389/fncom.2021.666131)
Supplement: Supplementary file 1 [file Data_Sheet_1.pdf]

Supplementary Material

1 Supplementary Figures

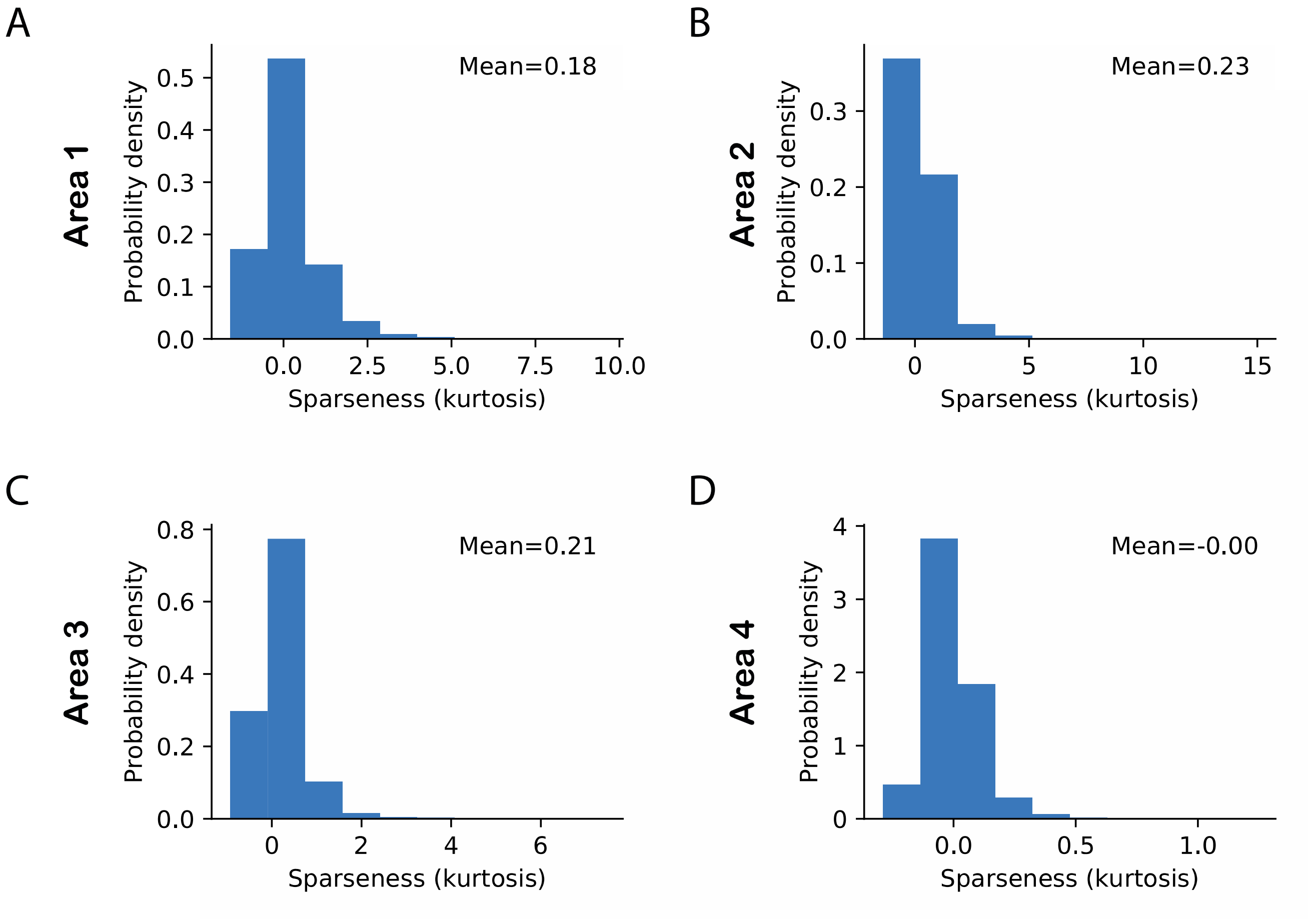

Supplementary Figure 1. Sparseness in neuronal activity across ascending areas in a linear model without regularization of weights and activity. Sparseness was measured as the kurtosis across all neuronal responses in a given area and given a single stimulus. The mean value of sparseness (top right corner) was computed by averaging these estimates of kurtosis across all stimuli. (A-D) Distribution of sparseness in each area. We used models with a linear activation function as exemplars of models without regularization because ReLu enforces neural activity to be always positive, thereby requiring a strong regularization penalty. In the absence of regularization, the average sparseness in the model increased modestly from areas 1 and 2 and then decreased in areas 3 and 4. Despite its modest effect size, this pattern was observed across multiple models with a varying number of areas. This is attributed to the network property that all areas in the model (except the top area) infer causes that reconcile bottom-up and top-down information (Equation 4 and 6) whereas causes in the top area are only determined by bottom-up information. The lower constraint on the top area leads to lower sparseness in this area. This effect was not limited to the top area alone;

it was generally applicable to areas in the hierarchy that were farther away from the sensory input layer.

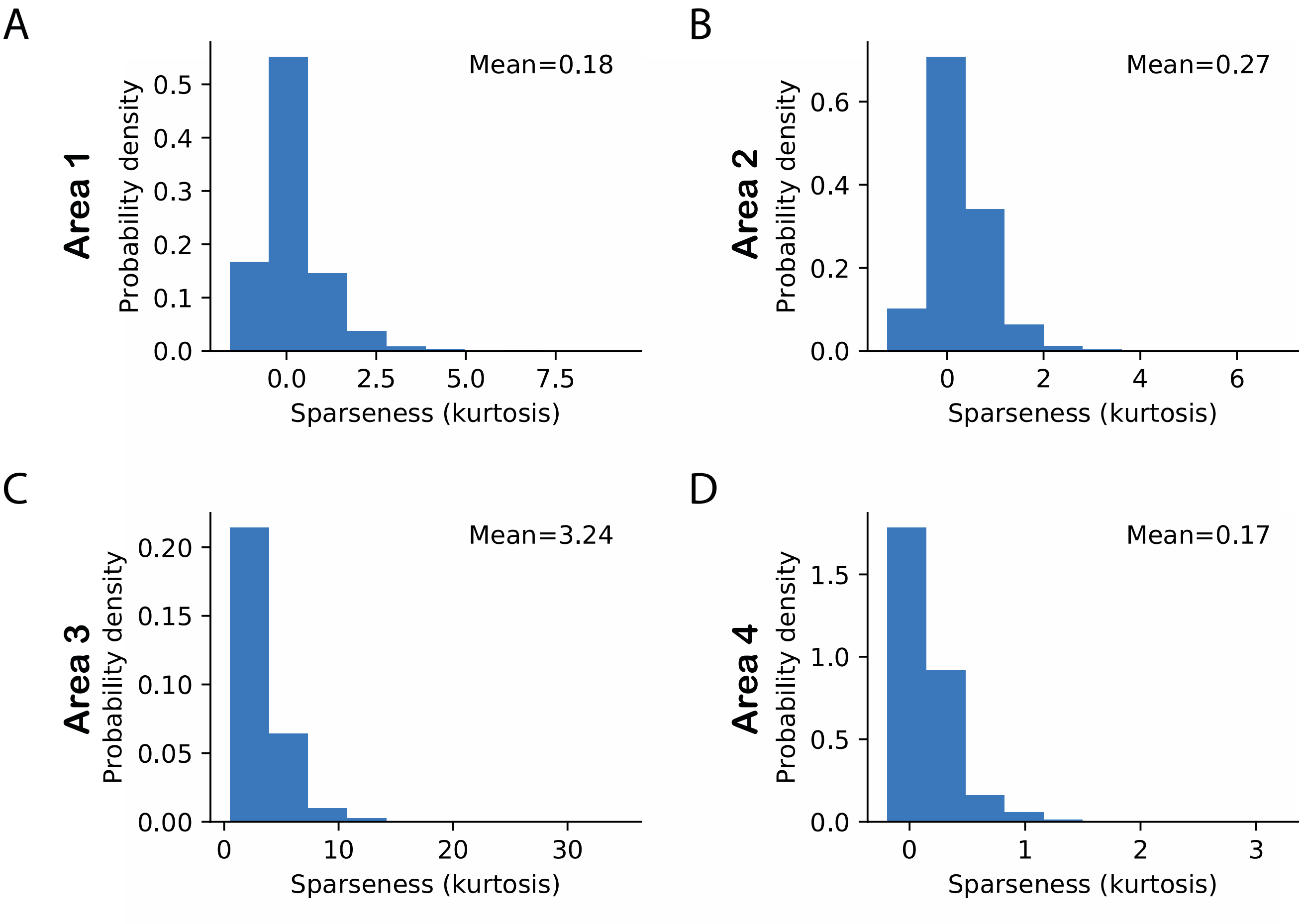

Supplementary Figure 2. Sparseness in neuronal activity across ascending areas in a linear model with regularization only in the top area. Sparseness was quantified as in fig. S1. The mean sparseness (top right corner) was computed by averaging these estimates of kurtosis across all stimuli. (A-D) Distribution of sparseness in each area. Having regularization only in the top area presents an interesting case because this indirectly regularizes all other model areas. Regularization-induced sparseness in area 4 results in sparse top-down predictions propagating to area 3, which indirectly induces sparseness in area 3 representations. Compared to Figure S1, regularization results in an increase in sparseness in area 4 and indirectly leads to an increase in sparseness in areas lower

than area 4. This effect is stronger in area 3 and becomes weaker as one moves away from the top area.

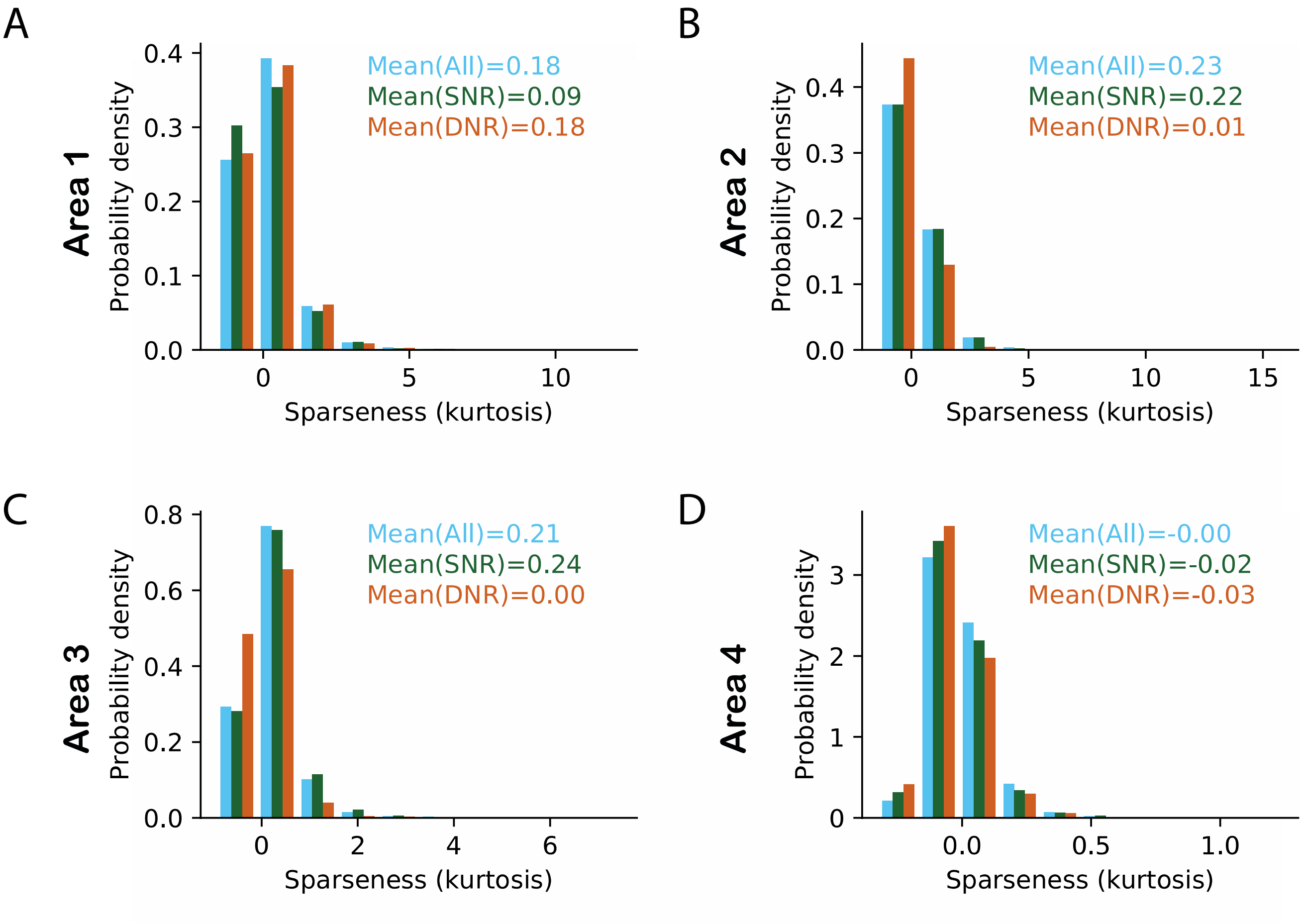

Supplementary Figure 3. Effect of high selectivity and high dynamic response range neurons on sparseness in a linear model with no regularization. (A-D) Histogram of sparseness for three different populations of neurons. The distribution of sparseness for all neurons has been shown in blue. The population in which the top 10% of most selective neurons was removed (SNR) is shown in dark green and light brown color denotes the populations in which neurons with high dynamic response range were removed (DNR). Values in top right corner represent mean sparseness estimates for the different populations in corresponding colors. It can be observed that high-selectivity neurons contribute to sparseness in the lowest area (area 1) whereas in areas 2 and 3 the high dynamic range neurons contribute to sparseness. Despite modest effect sizes, this pattern was observed across multiple model variants. The effects are attributed to the network property that area 1 receives a bottom-up input based on a fixed visual image. Other areas in the network receive a bottom-up drive based on a constantly evolving set of latent representations. This leads to higher dynamic ranges in

areas 2 to 3 and, as a result, sparseness is strongly determined by the dynamic response range in these areas.

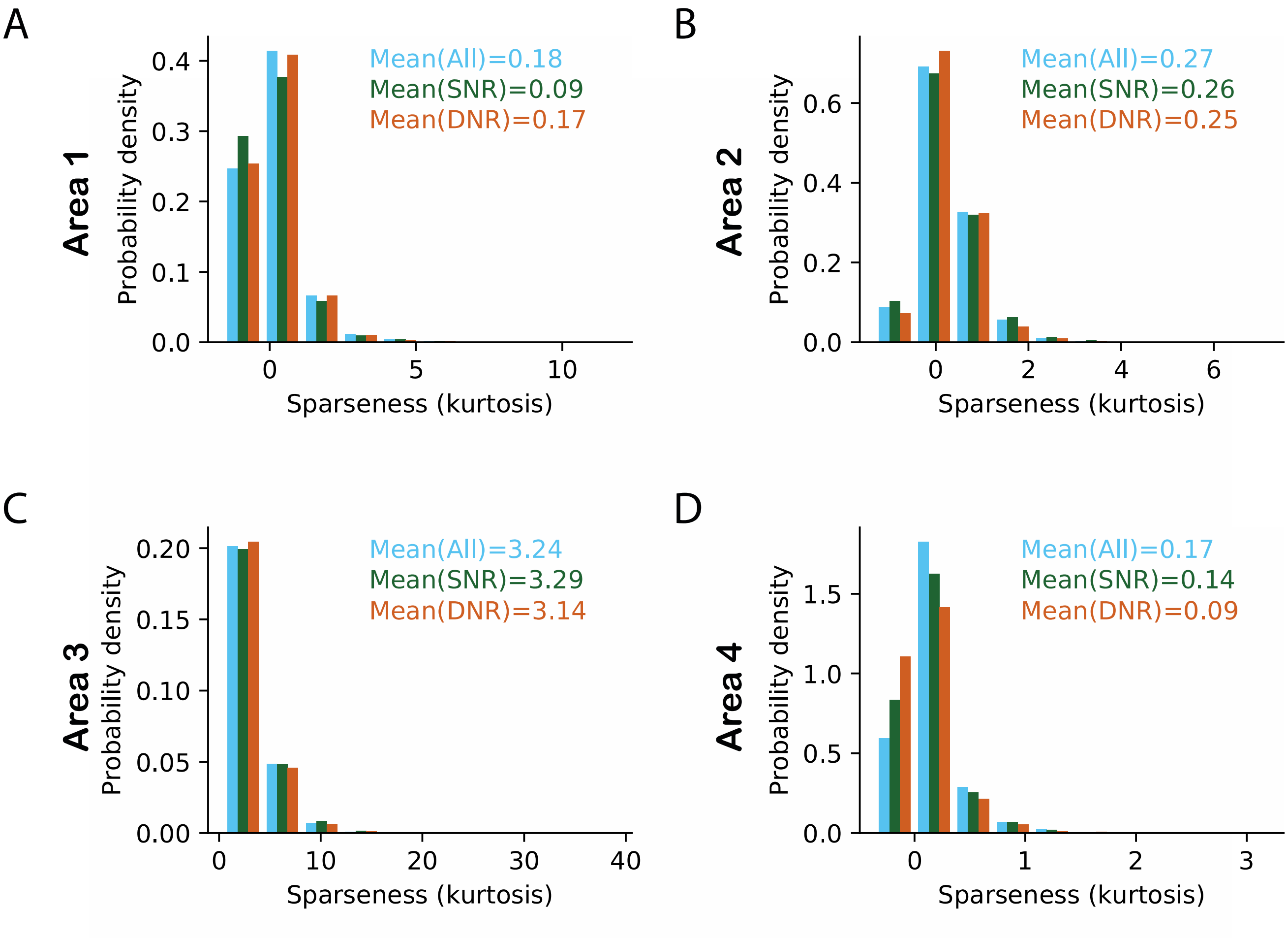

Supplementary Figure 4. Effect of high selectivity and high dynamic response range neurons on sparseness in a linear model with regularization only in the top area. (A-D) Histograms of sparseness for three different populations of neurons. The distribution of sparseness for all neurons is shown in blue. For plotting conventions, see figure S3. As a result of adding regularization to the top area, the contribution of high dynamic range neurons to sparseness is weakened in areas 2 and 3 (cf. Figure S3). This effect likely arises because regularization, by definition, reduces neuronal activity; via a top-down spreading effect this leads to lower dynamic ranges in areas 2 and 3. In turn, this reduces the contribution of high dynamic range neurons to sparseness in these areas.
